# A Global View to HBV Chronic Infection: Evolving Strategies for Diagnosis, Treatment and Prevention in Immunocompetent Individuals

**DOI:** 10.3390/ijerph16183307

**Published:** 2019-09-09

**Authors:** Laura Ambra Nicolini, Andrea Orsi, Paola Tatarelli, Claudio Viscoli, Giancarlo Icardi, Laura Sticchi

**Affiliations:** 1 Infectious Diseases Unit, IRCCS Policlinico San Martino Hospital, 16132 Genoa, Italy; 2 Hygiene Unit, IRCCS Policlinico San Martino Hospital, 16132 Genoa, Italy; 3 Department of Health Sciences, University of Genoa, 16132 Genoa, Italy; 4 UOC Malattie Infettive, Santa Maria delle Croci, 48121 Ravenna, Italy

**Keywords:** HBV chronic infection, HBV diagnosis, HBV prevention, HBV treatment, HBV vaccine

## Abstract

Hepatitis B Virus (HBV) is a significant public health challenge. Around 250 million people live with chronic HBV infection. With a global approach to this issue, we focus on new perspective in diagnosis, management and prevention of HBV chronic infection. Precise diagnosis of HBV status is crucial to guide patient management. Although available drugs reduce the risk of liver disease progression, they are not able to definitely eradicate HBV, and new therapeutic options are urgently needed. Thus, prevention of HBV infection is still the most effective strategy to achieve the control of the disease. Key aspects of prevention programs include surveillance of viral hepatitis, screening programs and immunization strategies. In spite of the high success rate of licensed HBV vaccines, a need for improved vaccine persists, especially in order to provide coverage of current non-responders.

## 1. Introduction

Hepatitis B Virus (HBV) is a significant public health challenge. Around 250 million people live with chronic HBV infection [1]. After several years from infection, approximately 15–40% of chronic infected patients develop serious sequelae such as cirrhosis, liver failure and hepatocellular carcinoma, and nearly one million people annually die due to HBV related complications [1,2]. 

Currently available antiviral treatment are able to persistently suppress viral replication and, in some cases, to obtain sustained off-treatment serological response [3], but definitive eradication of HBV infection is still an ideal outcome. The management of HBV infection is complicated even further by the emergence of HBV viral mutations able to confer resistance to antiviral agents, while the increasing incidence of HBV surface antigen (HBsAg) escape variants allows the virus to escape vaccine-induced immunity [4]. Additionally, approximately 5 of 100 healthy adults fail to achieve seroprotection after a full HBV vaccination course [5,6,7,8,9,10]. Thus, precise diagnosis is important to guide patient management and, together with prevention strategies, to control the spread of infection to susceptible individuals, while new therapeutic options are needed to achieve a complete cure of HBV infection. 

Based on this background, the aim of this review is to focus on the clinical impact of the most recent advances on diagnosis, prevention and treatment of HBV chronic infection in immunocompetent patients.

## 2. HBV Diagnostic Tools

HBV diagnosis and management is based on a combination of viral, host and liver disease factors. Indeed, HBV envelope Antigen (HBeAg) expression, HBV genotype, levels of viral replication (HBV DNA), HBV resistance mutations and stage of liver fibrosis should be acknowledged to select the best therapeutic approach. Beyond well-known diagnostic tools, significant advances in HBV diagnosis have been made in recent years, with a high potential for supporting treatment decision. Table 1 outlines blood tests currently recommended to guide starting and monitoring of HBV treatment. 

### 2.1. HBV Surface Antigen 

#### 2.1.1. HBsAg Escape Mutants 

Among serological markers, HBsAg is the hallmark of chronic infection, which is usually defined as the persistence of HBsAg in the blood for more than six months. Since the discovery of G145R mutation in 1988, evidences indicate that mutations in the major hydrophilic region (MHR) in the open reading frame (ORF) region, which encodes for HBsAg, and especially in the “a” determinant region (residue 124–147), may induce a conformational change of HBsAg, reducing its availability to specific HBs antibodies [11,12]. HBsAg escape mutants may derive from archived covalently closed circular DNA (cccDNA), and are mainly selected by pressure of HBV vaccination, especially in the case of plasma-derived vaccines, passive immunization with specific immunoglobulin (i.e., new-borns from carrier women and patients who underwent liver transplantation) and antiviral treatment, as in the case of lamivudine [13]. Besides selective treatment pressure-associated HBsAg mutants, natural immune-escape variants may develop among treatment naive chronic HBsAg carriers, leading to HBV viral replication even in presence of anti-HBs antibodies [13]. Moreover, Aragri et al. found a 53.2% prevalence of immune-escape mutation in a cohort of HBV acutely infected patients [14]. Of these mutations, sP120S, sM133L, and sG145R, known to act as vaccine-escape mutations, were detected in 11.4% of HBV genotype D-infected patients [14]. Molecular dynamic analysis revealed that the antigenic loops in the G145R mutant are not as exposed as those in the wild-type HBsAg, with a reduced immunogenic potential that may lead to vaccine escape [15]. Until now, a few cases of HBV infection with virus harboring MHR mutations among recipients of not-plasma derived vaccines have been reported, particularly in blood donors receiving HBV DNA testing before donations [16,17]. Thus, the possible impact of HBsAg escape mutants on vaccine efficacy should be acknowledged and taken into account. 

Another reason of concern regarding the emergence of HBsAg escape mutants is the risk of false negative HBsAg testing. Indeed, although manufacturers have improved assay ability to detect HBsAg escape mutants using a mixed monoclonal/polyclonal combination to minimize unexpected missing of HBsAg through epitope loss, results extremely vary [13]. Hossain et colleagues recently found that two natural amino acid mutations, lysine at 120 and aspartic acid at 123, were simultaneously responsible for the production of completely undetectable HBsAg (HBsAg with complete loss of antigenicity), when tested using commercial ELISA kits (Rapid II kit from Beacle Inc., Kyoto, and HBs ELISA Kit from Bioneovan Co., Ltd., Beijing, China) [18]. Hopefully, some new HBsAg assay allows earlier detection of vaccine breakthrough infections and more sensitive detection of HBsAg in the presence of anti-HBs [17]. HBV infection should be considered in patients with acute or chronic liver damage in the absence of an alternative diagnosis and despite HBV vaccination and/or negative HBsAg testing. 

#### 2.1.2. Quantitative HBsAg 

Quantification of HBsAg (qHBsAg) may be helpful for staging of HBV infection and prediction of response to antiviral therapy [19].

HBsAg levels inversely correlate with the degree of viral immune control and thus vary during the different phases of HBV infection [20]. qHBsAg depends on both viral and host factors, such as genotype, preS/S gene variability and hepatic disease stage (being higher in cirrhotic patients). Previous studies showed that qHBsAg may be helpful in the differential diagnosis between immune tolerance and immune reactive HBeAg positive phases in patients with high HBV DNA values but normal or slightly elevated alanine aminotransferase [21]. Additionally, qHBsAg supports the distinction between “true inactive HBsAg carriers” and HBeAg-negative HBV chronic hepatitis in HBsAg chronic carriers with negative HBeAg and HBV DNA below 2000 IU/mL [22,23]. qHBsAg higher than 1000 IU/mL may be helpful to identify those patients at high risk of developing hepatocellular carcinoma (HCC) despite HBV DNA below 20,000 UI/mL and negative HBeAg status [24]. 

For HBeAg-positive individuals, a decline below 1500 IU/mL of qHBsAg at week 12 of pegylated interferon (pegIFN)-based treatment is associated with sustained virological suppression and serological response, while qHBsAg above 20,000 IU/mL at weeks 12 and 24 in patients with genotype B and C is associated to lack of HBeAg loss [25,26,27,28]. Similarly, for HBeAg-negative patients with HBV genotype D infection, a decline of less than 2 log of qHBsAg at week 12 is associated with lack of sustained virological response [29,30]. Consequently, current guidelines states that testing qHBsAg before and during (particularly at weeks 12 and 24) treatment can be useful in managing patients receiving pegIFN therapy [31].

With regard to nucleoside analogues (NAs) treatment, qHBsAg decline was not related to on-treatment HBV DNA reduction although it could be associated to off-treatment virological response [32,33]. In HBV suppressed HBeAg-negative patients who discontinued entecavir (ETV), qHBsAg gradually decrease over a median period of 6–7 months and then precipitously decline 2–3 years prior to HBsAg loss [34]. At present, qHBsAg testing is not recommended in case of NAs treatment [31].

qHBsAg may also vary in presence of co-infection with viruses other than HBV. Indeed, qHBsAg is directly related to hepatitis Delta (HDV) viral load in HBV/HDV co-infected patients [35] and inversely related to CD4+ T cell recovery in case of HBV/HIV co-infection [36]. Given together, these evidences suggest that qHBsAg should be tested in selected patients and at precise time-point in order to tailor HBV treatment.

### 2.2. HBV Sequencing

Beyond the aforementioned risk related to HBsAg escape mutantions and the well-known risk of HBeAg negative hepatitis (via inhibition of HBeAg synthesis), HBV mutations may cause resistance to anti-HBV drugs and increase the risk for carcinogenesis.

The pol open reading frame (ORF) codes for transcriptase (RT) domain of HBV polymerase, which is the target for current available oral antiviral nucleoside analogues (NA). Naturally occurring mutations originate spontaneously from high error rates during replication (estimated to be about 1 for 105–107 nucleotide in the course of each replication cycle) and lack of proof reading during reverse transcription of pre-genomic RNA to HBV DNA [37]. Additionally, pol ORF mutations may be selected under drug pressure or as compensatory mutations following changes in the preS/S ORF [38].

According to current guidelines of the American Association for the Study of Liver Diseases (AASLD), HBV sequencing is usually not required in treatment naive patients, as mutation rates is usually below 5%, [31]. On the other hand, HBV resistance testing should be performed in case of past HBV treatment, virological failure and virological breakthrough during treatment (Table 1) [36]. Different assays for RT domain sequencing are currently available. Among genotypic assays, direct sequencing is the gold standard method, as it can detect all existing and emerging mutations that are present at significant proportions (i.e., at least 20% of the viral population) [39]. Other recently developed assays, including line probe assays and restriction fragment length polymorphism analyses, may detect minor viral species that are expressed at low proportion (i.e., <5%), but they are able to identify only already known viral mutations [39,40,41,42,43]. Additionally, Ionization Time-Of-Flight Mass Spectrometry based on mass spectrometric analysis of small DNA fragments containing sites of variation, can detect mutants that constitute lower than 1% of the total viral population [44]. Recently, more sensitive technologies such as ultradeep pyrosequencing have become available, but currently their use is not recommended in clinical practice [31]. Additionally, an in vitro phenotypic assay would be helpful to confirm genotypic antiviral resistance, although current methodology is labor-intensive and time consuming [45].

With regard to the risk of carcinogenesis, some deletion in preS1 and S2 regions and point mutations in the S region have been associated with the risk of HCC, via altered HBsAg secretion and intracellular accumulation of viral particles [36]. According to a meta-analysis including more than 11,000 patients, patients with preS mutants have a 3.8 fold increased risk of HCC [46]. Finally, a role for X ORF region in carcinogenesis has been postulated, but the strength of association is still under investigation [47].

### 2.3. cccDNA and HBV RNA

Both intracellular cccDNA and secreted HBV RNA point to the replicational activity of HBV [48,49]. cccDNA detection has some technical limitations, including complicated time-consuming tecniques, high costs and specificity concerns. On the other hand, accurate and sensitive tecniques are available for detecting HBV RNA in serum, a surrogate marker for cccDNA. Moreover, HBV RNA detection has a good correlation with HBeAg seroconversion in HBeAg positive patients receiving pegIFN.

## 3. HBV Treatment

### 3.1. Goals of HBV Treatment and Definitions of Response to Anti-HBV Treatment

The final goal of treatment of chronic HBV-related hepatitis is to reduce the risk of progression to cirrhosis and liver-related complications, including hepatocellular carcinoma (HCC) [31]. HBV DNA suppression (i.e., virological response) and HBeAg or HBsAg seroconversion, respectively in HBeAg positive and HBeAg negative chronic hepatitis (i.e., serological response), are currently used to measure the effectiveness of HBV treatment. However, despite virological and serological response strongly reduce the probability of cirrhosis and HCC, a residual risk still exists [50,51].

To completely prevent the risk of clinically significant consequences of HBV infection, some authors focused on the role of cccDNA in maintaining HBV persistence and cause progression of the liver disease [52]. Indeed, cccDNA is the template for all HBV mRNAs, and a few copies of cccDNA per liver are sufficient to (re) initiate HBV infection [53]. Thus, two novel definitions of HBV cure have been recently proposed. The former is functional HBV cure, that is sustained off-drug suppression of serum HBsAg and seroconversion to antibodies against HBV (HBsAb) in spite of persistence cccDNA in a transcriptionally inactive status. The latter is complete HBV cure, that is sustained off-drug seroconversion to HBsAb associated with eradication of cccDNA, with complete clearance of the virus from the body. [52]. Two diagnostic tools that we previously mentioned could be used to pinpoint complete cure, cccDNA and serum HBV RNA. Of note, HBV RNA can be readily detected by very accurate and sensitive methods that are widely used in diagnostic laboratories [49].

### 3.2. Currently Available Therapeutic Options: pegIFN and NA

Available classes of drugs against HBV include pegIFN and NAs. Both of them are able to achieve virological response and, in a minority of cases, serological response. Although they may partially suppress transcription of the already established cccDNA [54,55,56,57], they do not prevent the initial formation of cccDNA after de novo infection of hepatocytes [42]. Thus, while virological and serological responses are achievable endpoints, functional and complete cure cannot be attained with current available anti-HBV drugs. While reasons for cccDNA decrease during treatment with NAs are not entirely understood, pegIFN can directly mutate and destroy HBV cccDNA via induction of APOBEC deaminases [58,59].

#### 3.2.1. pegIFN

Although pegIFN may inhibit viral transcription independently of immune cells, it mainly acts as immune modulatory agent through cell-mediated immunity stimulation. Despite the well-known risk of adverse events and the need of subcutaneous administration, it is still an attractive therapeutic option because of the finite duration of treatment [31]. Indeed, following 48 weeks of treatment, up to 30% of HBeAg positive patients experience sustained off treatment seroconversion to antibodies against HBeAg (HBeAb) [58], whilst up to 4% of HBeAg negative patients develop HBsAg loss [58]. The identification of reliable predictors of response to pegIFN such as HBV genotype and liver fibrosis stage has also allowed identification of those patients with higher chances of response. Pre-treatment predictors of response to pegIFN include HBV genotypes A and B, low viral load, serum alanine aminotransferase (ALT) levels above 2–5 times normal values and high activity scores at liver fibrosis [50]. Moreover, as previously mentioned, on treatment qHBsAg levels have been used, together with HBV DNA decline and genotype, to design stopping rules for early pegIFN discontinuation in case of lack of response [31]. Additionally, HBV RNA could be used to predict response to pegIFN therapy [48,49].

Thus, careful identification of patients with higher response rates and prompt withdrawal of treatment according with stopping rules maximizes the benefits and minimizes the risks of pegIFN treatment.

#### 3.2.2. NAs

NAs specifically target the HBV reverse transcriptase and, consequently, inhibit the formation of progeny virus, with a low risk of adverse events. Among available NAs, tenofovir and ETV are currently used as first line therapeutic options because of satisfactory virological efficacy, high genetic barrier to virological resistance and excellent safety profile [31]. While tenofovir disoproxil fumarate (TDF) has been the only available formulation of tenofovir for a long while, tenofovir alafenamide (TAF) is a new prodrug recently released by the Food and Drug Administration, with better renal and bone safety profile and similar virological efficacy than TDF [59,60,61,62,63]. The high genetic barrier of TDF, TAF and ETV minimize the risk of virological failure, but genotypic testing is recommended in case of previous exposure to low genetic barrier NAs such as lamivudine, because of the risk of reduced susceptibility to ETV [64,65]. Among diagnostic tools, loss of RNA in subjects with undetectable HBV DNA could potentially serve as a new NA treatment endpoint alone or in conjunction with other markers [48,49].

Beyond virological response, high genetic barrier NAs are able to induce HBeAg and HBsAg loss in up to 40% and 12% of HBeAg positive and HBeAg negative patients, respectively, at 4 and 7 years of follow up [66,67]. Additionally, the longer the treatment duration, the higher the probability of serological response, as HBeAg loss rates increase from 17% at one year to 41% at four years of treatment [66]. However, as the majority of patients do not achieve serological response and withdrawal of NAs could result in relapse of viral infection, the length of treatment is life-long in the majority of cases.

### 3.3. New Therapeutic Options against HBV

When weighting up the inability of current anti-HBV drugs to achieve functional and complete HBV cure, the low safety profile of pegIFN and need of life-long treatment in case of NAs treatment and the residual risk of liver disease progression despite HBV-DNA suppression, it seems clear that new therapeutic options are urgently needed.

#### 3.3.1. New Therapeutic Options with Current Available Anti-HBV Drugs

In recent years, extending treatment duration of pegIFN as well as combination strategies have been investigated in order to enhance the efficacy of current available treatments. An Italian multicenter study demonstrated that extended treatment duration with pegIFN to 96 weeks improved the rates of sustained virological response (29% *vs.* 12%, *p* = 0.03) in HBeAg-negative genotype D patients when compared to 48 weeks of treatment, although seroconversion rates were similar, without impairing tolerability [68].

Additionally, several studies focused on NAs and pegIFN dual treatment, i.e., the association of these two therapeutic classes since the beginning of antiviral treatment, add-on and switch strategies. However, a few randomized trials in this setting are available, and current evidence is mainly based on open-label studies.

With regard to dual treatment with pegIFN and low genetic barriers DAAs (lamivudine, telbivudine, adefovir) the only available randomized trial at our knowledge evaluated combination therapy with pegIFN and TDF [69]. Authors compared four different arms: TDF plus pegIFN for 48 week, TDF plus pegIFN for 16 weeks followed by TDF for 32 weeks, TDF for 120 weeks and pegIFN for 48 weeks. HBsAg loss rates were higher in the combination arms, but among HBeAg negative patients near all cases of serological response have been registered among genotype A patients [69]. Thus, this result should be interpreted with caution.

Regarding add-on strategies, in the ARES study 24 weeks of pegIFN add-on therapy significantly increased HBeAg loss rates compared to ETV monotherapy, and it appeared to prevent relapse after stopping ETV, representing a promising strategy in selected patients [70]. In the PEGOS trial, HBeAg-positive patients with compensated liver who were treated with entecavir/tenofovir for at least 12 months and had an HBV DNA load of <2000 IU/mL disease were randomized to receive pegIFN add-on for 48 weeks or continue NA monotherapy. Authors found that the add-on strategy lead to higher rate of HBeAg seroconversion than NA monotherapy, though not statistically significant [71]. In HBeAg negative patients, pegIFN add-on therapy was associated with enhanced HBsAg decline, though HBsAg loss rates did not increase [72,73]. The impact of switching strategies from high barrier NA to pegIFN was evaluated by two randomized trials. In patients with baseline positive HBeAg, switching from ETV to a 48 weeks pegIFN course significantly increased serological response rates, and HBsAg loss rates were higher particolarly in case of baseline qHBsAg below 1500 UI/mL [74]. On the other hand, Xie et al. did not find a benefit of adding pegIFN add-on at week 13 of treatment with ETV or following 24 weeks of ETV compared with peg-IFN alfa-2a monotherapy [75].

Further studies are needed to assess the efficacy of these strategies.

#### 3.3.2. New Anti-HBV Drugs

Several new drugs targeting different steps of HBV life cycle are currently under investigation, including both molecules that directly target HBV and immunomodulators (Table 2). For some of them, phase III studies have been started or completed, whilst others have phase IIb results and, thus, are expected to become available in a few years.

Besifovir is an acyclic nucleotide phosphonate with similar chemical structure to TDF that has recently been approved in Korea [76]. A phase IIb trial evaluating 90 mg and 150 mg daily besifovir doses showed equal virological suppression rates than entecavir 0.5 mg daily in treatment-naïve chronic HBV patients [76]. Additionally, in a phase III trial comparing besifovir 150 mg or TDF 300 mg in chronic HBV patients, besifovir showed comparable virological efficacy and better histological response, in spite of reduced bone and renal toxicity, than TDF [77]. No studies addressed the effect of besifovir on cccDNA so far, but NAs usually do not prevent cccDNA initial formation; thus, it is improbable that besifovir could lead to functional and complete cure. Along with novel NAs, other antivirals under development are viral entry inhibitors, which include drugs targeting cccDNA formation and its epigenetic regulation, the HBx protein functions, nucleocapsid assembly and pgRNA packaging, viral DNA synthesis, and viral morphogenesis. As of today, only a few compounds have reached phase II clinical development. Among them, Bulevirtide (Myrcludex-B; Hepatera Ltd, Moscow, Russia) is a synthetic lipopeptide derived from the HBV envelope protein that inhibit HBV entry into the hepatocytes [78]. Bulevirtide blocks the receptor functions of the sodium taurocholate co-transporting polypeptide, which is usually bound by the myristoylated N-terminal preS1 domain of the HBV L protein to mediate attachment of virions to the surface of hepatocytes [78]. Preclinical studies investigated the ability of Bulevirtideof preventing primary HBV infection as well as intrahepatic viral spreading at a time when only a minority of the human hepatocytes are infected [79,80,81]. Particularly, Volz et al. did not find increasing in viremia, antigen-levels and amount of HBcAg-positive human hepatocytes, whilst ccDNA pool amplification was hindered in initially infected hepatocytes [81]. Additionally, whilst Bulevirtide seems not to affect HBsAg levels, it efficiently hinders the establishment of hepatitis Delta (HDV) infection in vivo in uPA/SCID mice, making it the first selective drug against HDV [80]. These data highlight the possible use of Bulevirtide in preventing HBV transmission, like in case of HBV and/or HDV exposure, prevention of HBV mother to child transmission and HBV-infected liver recipients, as well as in patients with HBV/HDV co-infection. Although phase IIb/III studies are still pending, Bulevirtide might increase the probability of achieving a functional cure for HBV [79]. Other anti-HBV drugs that completed phase II study are ARC-520 (Arrowhead Pharmaceutical, Inc., Pasadena, California, USA), a HBV specific small interfering RNA (siRNA), GS-9620, an immune modulator agonist of Toll like receptor-7 (TLR-7) and a therapeutic vaccine named ABX203/NASVAC.

Like other siRNA, ARC-520 induces a gene silence at a post-transcriptional level [82]. ARC-520 was evaluated as single-dose in ETV-naïve and experienced chronic HBV patients, showing a clear reduction of HBsAg levels, although no impact on cccDNA, in phase I trials [83]. The manufacturer has discontinued the development of ARC-520 and other siRNA that utilize a delivery vehicle called EX1, because of safety concerns during a non-clinical toxicology study in non-human primates. Importantly, regulators had never expressed any concern about clinical data [84].

GS-9620 is an oral agonist of toll-like receptor 7 that showed high tolerability and safety, but no significant HBsAg decline in chronic HBV patients who are suppressed on antiviral treatment [85]. Further evaluation is needed in patients not currently on HBV treatment.

Among therapeutic vaccines, ABX203/NASVAC is a new formulation combining HBV surface and core antigens, which has formerly shown superior efficacy compared with pegIFN alpha in treatment naïve chronic HBV patients [86]. However, a phase III trial evidenced that ABX203/NASVAC does not prevent viral relapse after stopping NAs [87]. Several other molecules targeting cccDNA (i.e., inhibitors of nucleocapsid assembly, rcDNA production and conversion to cccDNA, inhibitors of cccDNA maintenance and molecules that alter chemical structure of cccDNA) as well as immunotherapeutic strategies are currently on phase I of development. Thus, the HBV therapeutic scenario will be implemented in the next decade.

## 4. HBV Prevention Strategies

### 4.1. HBV Surveillance, Screening and Immunization Strategies

Surveillance of viral hepatitis, screening and immunization strategies are altogether key aspects of prevention programs for HBV infection.

The most frequent surveillance systems are based on national mandatory notification of HBV infection cases, although in a very few countries these can be classified as “active”, which means that the surveillance system is based on the initiative of public health officials to actively contact physicians, laboratory, hospital staff or other relevant sources to report data. Surveillance systems vary considerably throughout the world, and particularly in Europe, with differences between countries in terms of reporting practices, data collection methods and case definitions [88,89]. The continuous implementation of surveillance system allows scaling up of screening and immunizations programs, impacting the incidence of new cases of HBV infection. HBV screening and immunizations programs are currently based on a mix of different approaches and diversified among countries, depending on the epidemiology of the disease and the economic and resources condition (Table 3).

The majority of programs target selected populations, such as pregnant women and blood donors. Additionally, some countries provide HBV testing to specific risk groups, including people who inject drugs, prisoners, sex workers and, in some cases, to military recruits, people with multiple sexual partners and residents of long-term health facilities [90].

HBV immunization programs can be divided into universal and targeted. The universal approach, which is currently recommended by the WHO, typically utilizes routine childhood vaccination in combination with catch-up programs for older children and adolescents. [90,91]. The targeted approach focuses on individuals at high risk for HBV infection or HBV-related morbidity and mortality in reason of chronic diseases, lifestyle and occupation [92]. A few countries, mainly in low-prevalence and low endemicity areas, use a targeted approach as the only immunization strategy against HBV. Important limits affecting the targeted approach are the need to identify, test, and vaccinate all individuals at risk, as well as the low vaccine coverage rate achieved in the targeted populations [93,94,95,96,97]. Taking into account the changing profile of HBV epidemiology related to migration flows from intermediate/high to low endemicity countries, universal vaccination together with catch-up programs for people at risk is currently the preferred approach.

While HBV immunization represents the most meaningful point of prevention programs, data reviewed by WHO and UNICEF highlights that up to the 19% of infants worldwide did not receive the third dose of HBV vaccine [98]. Thus, the global health sector strategy on viral hepatitis indicated an increase in routine childhood hepatitis B virus vaccination coverage from 82% in 2015 to 90% by 2020 as an essential intervention [99]. Strengthening prevention programs will be necessary to achieve this goal.

### 4.2. HBV Vaccines: Current Available Strategies and Future Options

Technological advances in vaccinology allowed a substantial improvement from first generation HBV vaccines, based on purified virus from the plasma of asymptomatic human carriers [96], to second generation ones, which are based on yeast-derived recombinant hepatitis B surface antigen (rHBsAg) [7,100]. rHBsAg-based vaccines have similar success rate (up to 95% in case of healthy individuals) and a better safety profile than plasma derived. However, despite this marked improvement and the efficacy of universal and catch-up vaccination programs in reducing the incidence of HBV infection, a need for improved vaccine still exists, especially in order to enhance immunogenicity rates, to reduce the number of vaccine doses and to increase adherence to the vaccination schedule. Indeed, at present, approximately 5 of 100 healthy adults fail to achieve seroconversion (i.e., HBsAb higher than 10 mUI/mL), even after completing a full vaccine course, which requires the intramuscular administration of three vaccine doses at 0, 1 and 6 months [5,6,7,8,9,10].

Thus, different strategies have been evaluated to implement HBV vaccination. Several studies have focused on predictors of seroconversion after vaccination. Among them, the site and the route of injection, older age, obesity, renal failure, immunodeficiency and certain HLA types, particularly homozygosity for HLA-DRB1 * 0301 and HLA-DRB1 * 0701, have been associated with lack of immunogenicity [101,102,103]. Additionally, the timing, dosing and route of administration of rHBsAg-based vaccines have been studied. Accelerated schedules (four doses at 0, 1, 2, and 12 months, or four doses at 0, 7–10 days, 21 days, and 12 months) were proposed to improve adherence [104,105,106] and elicit a more rapid seroconversion than standard schedule, but long-term protection rates are largely unknown. At the same time, intradermal injection with lower doses of rHBsAg and double doses of rHBsAg have been studied among immune-compromised patients, showing higher response rates than standard schedule [107]. Consequently, British HIV Association (BHIVA) guidelines has implemented its vaccination policy, and 40-mcg intramuscular three-dose schedule is now the standard approach in the case of immunosuppression [108]. Much progress has also been made regarding the development of intranasal and oral delivery of rHBsAg vaccine, but optimization of such approaches are still needed [109]. Indeed, mucosal delivery of vaccines seems to induce a strong and effective mucosal immune response in a more efficient manner than parenteral delivery, which generally tends to induce only systemic immune response [110,111,112].

The use of novel adjuvants, alternative or additional to alum adjuvants, has increased immunogenicity of conventional HBsAg vaccines, particularly in case of products containing 3-deacylated monophosphoryl lipid A (3D-MPL), MF59, AS04 and synthetic oligodeoxynucleotides containing immunostimulatory CpG motifs that target TLR-9 and subsequently activate innate and adaptive immunity [113,114,115,116]. Other examples of these approaches are the combination of 3D-MPL and aluminum adjuvant, called SmithKline Beecham Adjuvant System 4; an adjuvant composition consisting of saponin QS21, 3D-MPL and an oil-in-water emulsion comprising squalene, tocopherol, cholesterol, and polysorbate 80; combinations of adjuvants (i.e., 3D-MPL, QS21, and CpG oligonucleotide) adsorbed to the phosphate or hydroxide of aluminum, zinc, calcium, cerium, chromium, iron or beryllium [117,118]. Furthermore, other molecules have been described or claimed for use as an adjuvant for HBV vaccine, in particular a combination of single-strand deoxynucleotides and one or more CpG dinucleotides, various synthetic peptides, an immunogenic complex termed ISCOM (for Immune Stimulating Complexes), a saponin adjuvant (AbISCO-200) [115].

In February 2018 the Advisory Committee on Immunization Practices (ACIP) recommended a yeast-derived vaccine prepared with a synthetic immunostimulatory cytidine-phosphate-guanosine oligodeoxynucleotide adjuvant, administered as a 2-dose series (0 and 1 month) for use among adults aged ≥18 years [119].

Finally, novel antigens alternatives to rHBsAg have been explored, although the clinical success and the commercial acceptance of conventional recombinant HBV vaccine have limited the interest in this field. Among new antigens, some authors have exploited the pre-S1 and pre-S2 surface proteins of HBV, which have been shown to play an important role in immunogenicity against HBV in both genetically resistant mice and in humans [120]. As of today, new recombinant HBV vaccines containing the pre-S1 and pre-S2 envelope antigens, produced using Chinese hamster ovary cells, have been licensed in a few countries [121,122,123].

### 4.3. HBV Treatment as Prevention

The potential role of some NA for pre-exposure prophylaxis has been evaluated among people at high risk for HBV infection, including men who have sex with men and HIV infected people. Studies exploring the epidemiology of HBV infection in previously unvaccinated or non-responders to vaccination patients showed lower risk of HBV infection in case of prophylaxis with lamivudine (3TC) or tenofovir (TDF) [124,125]. By contrast, Falade-Nwulia et al. did not find a protective role of prophylaxis over vaccination [126]. Thus, the benefit of HBV prophylaxis is unclear and does not seem cost-effective.

## 5. Conclusions

Recent advances in diagnosis, treatment and prevention of HBV contribute altogether to achieve the goal of eliminating HBV worldwide.

Genotyping HBV mutants have become key steps to delineate patterns of resistance and, together with qHBsAg, are crucial for tailoring antiviral therapy, currently available anti-HBV drugs are able to control HBV replication and reduce the risk of liver disease progression, but not to definitely cure HBV. Among new anti-HBV drugs on development, besifovir and myrcludex-B will be available in a few years, the latter of whom might also be able to achieve functional cure. Waiting for new therapeutic advances, prevention of HBV infection still remains the most effective strategy to achieve the control of the disease. Licensed HBV prophylactic vaccines are very effective against HBV infection, but low coverage rates in high-prevalence countries and difficulty reaching high-risk individuals represent obstacles that should be addressed in the next future.

## Figures and Tables

**Table 1:** Selected diagnostic tests used in management of HBV according to American Association for the Study of Liver Diseases (AASLD) guidelines (36).

Diagnostic test	Timing	pegIFN	NAs Treatment (TDF, TAF, ETV)
Quantitative HBV DNA	before tx	yes	yes
	during/after tx	yes	yes
HBV genotyping		yes	no
HBV sequencing	before tx	no	no
	during/after tx	no	selected cases *
qHBsAg	before tx	yes *	no
	during tx	yes *	no

* See text for details. Abbreviations: pegIFN, pegylated Interferon; TDF, tenofovir disiproxil; TAF, tenofovir alafenamide; ETV, entecavir; tx, treatment; HBV DNA, HBV viral load; qHBsAg quantitative HBsAg.

**Table 2:** New anti-HBV drugs with phase II/III study results and their mechanism of action.

Anti-HBV Drug	Mechanism of Action
besifovir	nucleoside analogue
bulevirtide	viral entry inhibitor
ARC-520	small interfereng RNA
GS-9620	Toll like receptor-7
ABX203/NASVAC	therapeutic vaccine

**Table 3:** Prevention strategies for HBV infection control in relation to available cost-effectiveness evaluations.

Strategy	Country Characteristics		
	Low- and Intermediate-Income Countries High HBV Prevalence ^a^	High- and Intermediate-Income Countries Intermediate HBV Prevalence ^b^	High- and Intermediate-Income Countries Low HBV Prevalence ^c^
Passive surveillance systems	√	√	√
Active surveillance systems		√	√
Screening program of any type	√	√	√
Screening program for specific risk groups only	√		
Screening program for pregnant women		√	√
Combined screening program			√
Use of hepatitis B immune globulin		√	√
Universal vaccination alone	√		
Universal vaccination + pregnant women screening + hepatitis B immune globulin		√	
Selective vaccination + pregnant women screening + hepatitis B immune globulin			√

^a^ Southeast Asia, China, Pacific islands, Sub-Saharan Africa, Alaska (Eskimos); ^b^ Mediterranean basin, Eastern Europe, Central Asia, Japan, Latin and South America, Middle East; ^c^ United States, Canada, Central Asia, Western Europe, Australia, New Zealand.

## References

[1] (2017). Global Hepatitis Report.

[2] Datta S., Chatterjee S., Veer V., Chakravarty R. (2012). Molecular biology of the hepatitis B virus for clinicians. J. Clin. Exp. Hepatol..

[3] Blumberg B.S. (1997). Hepatitis B virus, the vaccine, and the control of primary cancer of the liver. Proc. Natl. Acad. Sci. USA.

[4] Bottero J., Boyd A., Lemoine M., Carrat F., Gozlan J., Collignon A., Boo N., Dhotte P., Varsat B., Muller G. (2014). Current state of and needs for hepatitis B screening: Results of a large screening study in a low-prevalent, metropolitan region. PLoS ONE.

[5] Zuckerman J.N., Sabin C., Craig F.M., Williams A., Zuckerman A.J. (1997). Immune response to a new hepatitis B vaccine in healthcare workers who had not responded to standard vaccine: Randomised double blind dose-response study. BMJ.

[6] Marcus-Bagley D., Katz A.J., Brink S.J., Awdeh Z., Alper C.A., Kruskall M.S., Craven D.E., Dienstag J.L., Yunis E.J. (1989). Genetic prediction of nonresponse to hepatitis B vaccine. N. Engl. J. Med..

[7] McDermott A.B., Zuckerman J.N., Sabin C.A., Marsh S.G., Madrigal J.A. (1997). Contribution of human leukocyte antigens to the antibody response to hepatitis B vaccination. Tissue Antigens.

[8] Li Y., Ni R., Song W., Shao W., Shrestha S., Ahmad S., Cunningham C.K., Flynn P.M., Kapogiannis B.G., Wilson C.M. (2009). Clear and independent associations of several HLA-DRB1 alleles with differential antibody responses to hepatitis B vaccination in youth. Hum. Genet..

[9] Hennig B.J., Fielding K., Broxholme J., Diatta M., Mendy M., Moore C., Pollard A.J., Rayco-Solon P., Sirugo G., Van Der Sande M.A. (2008). Host genetic factors and vaccine-induced immunity to hepatitis B virus infection. PLoS ONE.

[10] Höhler T., Reuss E., Evers N., Dietrich E., Rittner C., Freitag C.M., Vollmar J., Schneider P.M., Fimmers R. (2002). Differential genetic determination of immune responsiveness to hepatitis B surface antigen and to hepatitis A virus: A vaccination study in twins. Lancet.

[11] Zanetti A.R., Tanzi E., Manzillo G., Maio G., Sbreglia C., Caporaso N., Thomas H., Zuckerman A.J. (1988). Hepatitis B variant in Europe. Lancet..

[12] Gerlich W.H., Glebe D., Schüttler C.G. (2007). Deficiencies in the standardization and sensitivity of diagnostic tests for hepatitis B virus. J. Viral Hepat..

[13] Alavian S.M., Carman W.F., Jazayeri S.M. (2013). HBsAg variants: Diagnostic-escape and diagnostic dilemma. J. Clin. Virol..

[14] Aragri M., Alteri C., Battisti A., Di Carlo D., Minichini C., Sagnelli C., Bellocchi M.C., Pisaturo M.A., Starace M., Armenia D. (2016). Multiple Hepatitis B Virus (HBV) Quasispecies and Immune-Escape Mutations Are Present in HBV Surface Antigen and Reverse Transcriptase of Patients with Acute Hepatitis, B. J. Infect. Dis..

[15] Rezaee R., Poorebrahim M., Najafi S., Sadeghi S., Pourdast A., Alavian S.M., Alavian S.E., Poortahmasebi V. (2016). Impacts of the G145R Mutation on the Structure and Immunogenic Activity of the Hepatitis B Surface Antigen: A Computational Analysis. Hepat. Mon..

[16] Luongo M., Critelli R., Grottola A., Gitto S., Bernabucci V., Bevini M., Vecchi C., Montagnani G., Villa E. (2015). Acute hepatitis B caused by a vaccine-escape HBV strain in vaccinated subject: Sequence analysis and therapeutic strategy. J. Clin. Virol..

[17] Kuhns M.C., McNamara A.L., Holzmayer V., Cloherty G.A. (2019). Molecular and serological characterization of hepatitis B vaccine breakthrough infections in serial samples from two plasma donors. Virol. J..

[18] Hossain M.G., Ueda K. (2017). Investigation of a Novel Hepatitis B Virus Surface Antigen (HBsAg) Escape Mutant Affecting Immunogenicity. PLoS ONE.

[19] Lee J.M., Ahn S.H. (2011). Quantification of HBsAg: Basic virology for clinical practice. World J. Gastroenterol..

[20] Nguyen T., Thompson A.J., Bowden S., Croagh C., Bell S., Desmond P.V., Levy M., Locarnini S.A. (2010). Hepatitis B surface antigen levels during the natural history of chronic hepatitis B: A perspective on Asia. J. Hepatol..

[21] Chan H.L., Thompson A., Martinot-Peignoux M., Piratvisuth T., Cornberg M., Brunetto M.R., Tillmann H.L., Kao J.H., Jia J.D., Wedemeyer H. (2011). Hepatitis B surface antigen quantification: Why and how to use it in 2011—A core group report. J. Hepatol..

[22] Brunetto M.R., Oliveri F., Colombatto P., Moriconi F., Ciccorossi P., Coco B., Romagnoli V., Cherubini B., Moscato G., Maina A.M. (2010). Hepatitis B surface antigen serum levels help to distinguish active from inactive hepatitis B virus genotype D carriers. Gastroenterology.

[23] Martinot-Peignoux M., Lada O., Cardoso A.C., Boyer N., Ripault M.P., Asselah T., Marcellin P. (2010). Quantitative HBsAg: A new specific marker for the diagnosis of HBsAg inactive carriage. Hepatology.

[24] Tseng T.C., Liu C.J., Chen C.L., Yang H.C., Su T.H., Wang C.C., Yang W.T., Kuo S.F.T., Liu C.H., Chen P.J. (2013). Risk stratification of hepatocellular carcinoma in hepatitis B virus e antigen-negative carriers by combining viral biomarkers. J. Infect. Dis..

[25] Tangkijvanich P., Komolmit P., Mahachai V., Sa-nguanmoo P., Theamboonlers A., Poovorawan Y. (2009). Low pretreatment serum HBsAg level and viral mutations as predictors of response to Peginterferon alfa-2b therapy in chronic hepatitis, B. J. Clin. Virol..

[26] Lau G., Marcellin P., Brunetto M. (2009). On treatment monitoring of HBsAg levels to predict response to peginterferon alfa-2a in patients with HBeAg-positive chronic hepatitis, B. J. Hepatol..

[27] Gane E., Jia J., Han K., Tanwandee T., Chuang W.L., Marcellin P., Chan H.L., Piratvisuth T., Wat C., Martins E. (2011). NEPTUNE study: On-treatment HBsAg level analysis confirms prediction of response observed in phase 3 study of peginterferon alfa-2a in HBeAg-positive patients. J. Hepatol..

[28] Sonneveld M.J., Hansen B.E., Piratvisuth T., Jia J.D., Zeuzem S., Gane E., Liaw Y.F., Xie Q., Heathcote E.J., Chan H.L.Y. (2013). Response-guided peginterferon therapy in hepatitis B e antigen-positive chronic hepatitis B using serum hepatitis B surface antigen levels. Hepatology.

[29] Cornberg M., Wong V.W., Locarnini S., Brunetto M., Janssen H.L., Chan H.L. (2017). The role of quantitative hepatitis B surface antigen revisited. J. Hepatol..

[30] Rijckborst V., Hansen B.E., Cakaloglu Y., Ferenci P., Tabak F., Akdogan M., Simon K., Akarca U.S., Flisiak R., Verhey E. (2010). Early on-treatment prediction of response to peginterferon alfa-2a for HBeAg-negative chronic hepatitis B using HBsAg and HBV DNA levels. Hepatology.

[31] Terrault N.A., Lok A.S.F., McMahon B.J., Chang K.M., Hwang J.P., Jonas M.M., Brown R.S., Bzowej N.H., Wong J.B. (2018). Update on Prevention, Diagnosis, and Treatment of Chronic Hepatitis B: AASLD 2018 Hepatitis B Guidance. Hepatology.

[32] Manesis E.K., Hadziyannis E.S., Angelopoulou O.S., Hadziyannis S.J. (2007). Prediction of treatment-related HBsAg loss in HBeAg-negative chronic hepatitis B: A clue from serum HBsAg levels. Antivir. Ther..

[33] Brunetto M.R., Moriconi F., Bononi F., Lau G.K., Farci P., Yurdaydin C., Wolf E. (2009). Hepatitis B virus surface antigen levels: A guide to sutained response to peginterferon alfa-2a in HBeAgnegative chronic hepatitis, B. Hepatology.

[34] Jeng W.J., Chang M.L., Liaw Y.F. (2019). Off-therapy precipitous HBsAg decline predicts HBsAg loss after finite entecavir therapy in HBeAg-negative patients. J. Viral. Hepat..

[35] Zachou K., Yurdaydin C., Drebber U., Dalekos G.N., Erhardt A., Cakaloglu Y., Degertekin H., Gurel S., Zeuzem S., Bozkaya H. (2010). HIDT-1 Study Group. Quantitative HBsAg and HDV-RNA levels in chronic delta hepatitis. Liver Int..

[36] Jaroszewicz J., Reiberger T., Meyer-Olson D., Mauss S., Vogel M., Ingiliz P., Payer B.A., Stoll M., Manns M.P., Schmidt R.E. (2012). Hepatitis B surface antigen concentrations in patients with HIV/HBV coinfection. PLoS ONE.

[37] Caligiuri P., Cerruti R., Icardi G., Bruzzone B. (2016). Overview of hepatitis B virus mutations and their implications in the management of infection. World J. Gastroenterol..

[38] Torresi J. (2002). The virological and clinical significance of mutations in the overlapping envelope and polymerase genes of hepatitis B virus. J. Clin. Virol..

[39] Pawlotsky J.M., Dusheiko G., Hatzakis A., Lau D., Lau G., Liang T.J., Locarnini S., Martin P., Richman D.D., Zoulim F. (2008). Virologic monitoring of hepatitis B virus therapy in clinical trials and practice: Recommendations for a standardized approach. Gastroenterology.

[40] Lok A.S.F., Zoulim F., Locarnini S., Mangia A., Niro G., Decraemer H., Maertens G., Hulstaert F., De Vreese K., Sablon E. (2002). Monitoring drug resistance in chronic hepatitis B virus (HBV)-infected patients during lamivudine therapy: Evaluation of performance of INNO-LiPA HBV DR assay. J. Clin. Microbiol..

[41] Zoulim F., Locarnini S. (2009). Hepatitis B virus resistance to nucleos (t) ide analogues. Gastroenterology.

[42] Valsamakis A. (2007). Molecular testing in the diagnosis and management of chronic hepatitis, B. Clin. Microbiol. Rev..

[43] Lok A.S., Zoulim F., Locarnini S., Bartholomeusz A., Ghany M.G., Pawlotsky J.M., Mizokami M., Kuiken C. (2007). Antiviral drug-resistant HBV: Standardization of nomenclature and assays and recommendations for management. Hepatitis B Virus Drug Resistance Working Group. Hepatology.

[44] Hong S.P., Kim N.K., Hwang S.G., Chung H.J., Kim S., Han J.H., Kim H.T., Rim K.S., Kang M.S., Yoo W. (2004). Detection of hepatitis B virus YMDD variants using mass spectrometric analysis of oligonucleotide fragments. J. Hepatol..

[45] Kim J.H., Park Y.K., Park E.S., Kim K.H. (2014). Molecular diagnosis and treatment of drug-resistant hepatitis B virus. World J. Gastroenterol..

[46] Liu S., Zhang H., Gu C., Yin J., He Y., Xie J., Cao G. (2009). Associations between hepatitis B virus mutations and the risk of hepatocellular carcinoma: A meta-analysis. J. Natl. Cancer Inst..

[47] Tang S., Hu W., Hu J., Wu S., Li J., Luo Y., Cao M., Zhou H., Jiang X. (2015). Hepatitis B virus X protein promotes P3 transcript expression of the insulin-like growth factor 2 gene via inducing hypomethylation of P3 promoter in hepatocellular carcinoma. Liver Int..

[48] Kostyusheva A., Kostyushev D., Brezgin S., Volchkova E., Chulanov V. (2018). Clinical Implications of Hepatitis B Virus RNA and Covalently Closed Circular DNA in Monitoring Patients with Chronic Hepatitis B Today with a Gaze into the Future: The Field Is Unprepared for a Sterilizing Cure. Genes.

[49] Cloherty G., Butler E., Kuhns M. (2019). Serum Hepatitis B Virus RNA as a Potential Diagnostic Biomarker During Chronic Hepatitis B Virus Infection. Clin. Liver Dis..

[50] Zhang W., Wang X., Wang Y., Zhao X., Duan W., Wang Q., Wu X., Kong Y., Ma H., You H. (2017). Effective viral suppression is necessary to reduce hepatocellular carcinoma development in cirrhotic patients with chronic hepatitis B: Results of a 10-year follow up. Medicine.

[51] Lin C.L., Kao J.H. (2013). Risk stratification for hepatitis B virus related hepatocellular carcinoma. J. Gastroenterol. Hepatol..

[52] Ning Q., Wu D., Wang G.Q., Ren H., Gao Z.L., Hu P., Han M.F., Wang Y., Zhang W.H., Lu F.M. (2019). Roadmap to functional cure of chronic hepatitis B: An expert consensus. J. Viral. Hepat..

[53] Nassal M. (2015). HBV cccDNA: Viral persistence reservoir and key obstacle for a cure of chronic hepatitis, B. Gut.

[54] Delmas J., Schorr O., Jamard C., Gibbs C., Trépo C., Hantz O., Zoulim F. (2002). Inhibitory effect of adefovir on viral DNA synthesis and covalently closed circular DNA formation in duck hepatitis B virus-infected hepatocytes in vivo and in vitro. Antimicrob. Agents Chemother..

[55] Le Guerhier F., Pichoud C., Jamard C., Guerret S., Chevallier M., Peyrol S., Hantz O., King I., Trepo C., Cheng Y.C. (2001). Antiviral activity of beta-L-2’,3’-dideoxy-2’,3’-didehydro-5-fluorocytidine in woodchucks chronically infected with woodchuck hepatitis virus. Antimicrob. Agents Chemother..

[56] Le Guerhier F., Pichoud C., Guerret S., Chevallier M., Jamard C., Hantz O., Li X.-Y., Chen S.-H., King I., Trepo C. (2000). Characterization of the antiviral effect of 2’,3’-dideoxy-2’, 3’-didehydro-beta-L-5-fluorocytidine in the duck hepatitis B virus infection model. Antimicrob. Agents Chemother..

[57] Werle–Lapostolle B., Bowden S., Locarnini S., Wursthorn K., Petersen J., Lau G., Trepo C., Marcellin P., Goodman Z., Iv W.E.D. (2004). Persistence of cccDNA during the natural history of chronic hepatitis B and decline during adefovir dipivoxil therapy. Gastroenterology.

[58] Allweiss L., Dandri M. (2017). The Role of cccDNA in HBV Maintenance. Viruses.

[59] Lucifora J., Xia Y., Reisinger F., Zhang K., Stadler D., Cheng X., Sprinzl M.F., Koppensteiner H., Makowska Z., Volz T. (2014). Specific and nonhepatotoxic degradation of nuclear hepatitis B virus cccDNA. Science.

[60] Moucari R., Martinot-Peignoux M., Mackiewicz V., Boyer N., Ripault M.P., Castelnau C., Leclere L., Dauvergne A., Valla D., Vidaud M. (2009). Influence of genotype on hepatitis B surface antigen kinetics in hepatitis B e antigen-negative patients treated with pegylated interferon-alpha2a. Antivir. Ther..

[61] Center for Drug Evalution and Research. https://www.accessdata.fda.gov/drugsatfda_docs/nda/2016/208464Orig1s000Approv.pdf.

[62] Buti M., Gane E., Seto W.K., Chan H.L.Y., Chuang W.-L., Stepanova T., Hui A.J., Lim Y.S., Mehta R., A Janssen H.L. (2016). Tenofovir alafenamide versus tenofovir disoproxil fumarate for the treatment of patients with HBeAg-negative chronic hepatitis B virus infection: A randomised, double-blind, phase 3, non-inferiority trial. Lancet Gastroenterol. Hepatol..

[63] Chan H.L.Y., Fung S., Seto W.K., Chuang W.-L., Chen C.-Y., Kim H.J., Hui A.J., A Janssen H.L., Chowdhury A., Tsang T.Y.O. (2016). Tenofovir alafenamide versus tenofovir disoproxil fumarate for the treatment of HBeAg-positive chronic hepatitis B virus infection: A randomised, double-blind, phase 3, non-inferiority trial. Lancet Gastroenterol. Hepatol..

[64] Tenney D.J., Levine S.M., Rose R.E., Walsh A.W., Weinheimer S.P., Discotto L., Plym M., Pokornowski K., Yu C.F., Angus P. (2004). Clinical emergence of entecavir-resistant hepatitis B virus requires additional substitutions in virus already resistant to Lamivudine. Antimicrob. Agents Chemother..

[65] Baldick C.J., Tenney D.J., Mazzucco C.E., Eggers B.J., Rose R.E., Pokornowski K.A., Yu C.F., Colonno R.J. (2008). Comprehensive evaluation of hepatitis B virus reverse transcriptase substitutions associated with entecavir resistance. Hepatology.

[66] Buti M., Tsai N., Petersen J., Flisiak R., Gurel S., Krastev Z., Schall R.A., Flaherty J.F., Martins E.B., Charuworn P. (2015). Seven-Year Efficacy and Safety of Treatment with Tenofovir Disoproxil Fumarate for Chronic Hepatitis B Virus Infection. Dig. Dis. Sci..

[67] Idilman R., Gunsar F., Koruk M., Keskin O., Meral C.E., Gulsen M., Elhan A.H., Akarca U.S., Yurdaydin C. (2015). Long-term entecavir or tenofovir disoproxil fumarate therapy in treatment-naïve chronic hepatitis B patients in the real-world setting. J. Viral Hepat..

[68] Lampertico P., Viganò M., Di Costanzo G.G., Sagnelli E., Fasano M., Di Marco V., Boninsegna S., Farci P., Fargion S., Giuberti T. (2013). Randomised study comparing 48 and 96 weeks peginterferon α-2a therapy in genotype D HBeAg-negative chronic hepatitis, B. Gut.

[69] Marcellin P., Ahn S.H., Ma X., Caruntu F.A., Tak W.Y., Elkashab M., Chuang W.-L., Lim S.-G., Tabak F., Mehta R. (2016). Combination of tenofovir disoproxil fumarate and peginterferon alpha-2a increases loss of hepatitis B surface antigen in patients with chronic hepatitis, B. Gastroenterology.

[70] Brouwer W.P., Xie Q., Sonneveld M.J., Zhang N., Zhang Q., Tabak F., Streinu-Cercel A., Wang J.-Y., Idilman R., Reesink H.W. (2015). Adding pegylated interferon to entecavir for hepatitis B e antigen-positive chronic hepatitis B: A multicenter randomized trial (ARES study). Hepatology.

[71] Chi H., Hansen B.E., Guo S., Zhang N.P., Qi X., Chen L., Guo Q., Arends P., Wang J.-Y., Verhey E. (2017). Pegylated Interferon Alfa-2b Add-on Treatment in Hepatitis B Virus Envelope Antigen-Positive Chronic Hepatitis B Patients Treated with Nucleos(t)ide Analogue: A Randomized, Controlled Trial (PEGON). J. Infect. Dis..

[72] Lampertico P., Brunetto M., Craxì A., Gaeta G., Rizzetto M., Palmieri G., Colombo M. (2015). LP25: Add-on peginterferon ALFA-2A significantly reduces hbsag levels in hbeag-negative, genotype d chronic hepatitis b patients fully suppressed on nucleot(s)ide analogue treatment: The HERMES study. J. Hepatol..

[73] Bourlière M., Rabiega P., Ganne-Carrie N., Serfaty L., Marcellin P., Barthe Y., Thabut D., Guyader D., Hezode C., Picon M. (2017). Effect on HBs antigen clearance of addition of pegylated interferon alfa-2a to nucleos(t)ide analogue therapy versus nucleos(t)ide analogue therapy alone in patients with HBe antigen-negative chronic hepatitis B and sustained undetectable plasma hepatitis B virus DNA: A randomised, controlled, open-label trial. Lancet Gastroenterol. Hepatol..

[74] Ning Q., Han M., Sun Y., Jiang J., Tan D., Hou J., Tang H., Sheng J., Zhao M. (2014). Switching from entecavir to PegIFN alfa-2a in patients with HBeAg-positive chronic hepatitis B: A randomised open-label trial (OSST trial). J. Hepatol..

[75] Xie Q., Zhou H., Bai X., Wu S., Chen J.J., Sheng J., Xie Y., Chen C., Chan H.L., Zhao M. (2014). A randomized, open-label clinical study of combined pegylated interferon Alfa-2a (40KD) and entecavir treatment for hepatitis B “e” antigen-positive chronic hepatitis, B. Clin. Infect. Dis..

[76] Lai C.L., Ahn S.H., Lee K.S., Um S.H., Cho M., Yoon S.K., Lee J.W., Park N.H., Kweon Y.O., Sohn J.H. (2014). Phase IIb multicentred randomised trial of besifovir (LB80380) versus entecavir in Asian patients with chronic hepatitis, B. Gut.

[77] Ahn S., Kim W., Jung Y., Yang J., Jang J., Kweon Y., Cho Y., Kim Y., Hong G., Kim D. (2017). Safety and efficacy of besifovir in treatment-naïve chronic hepatitis B virus infection: A randomized, double-blind, double dummy, phase 3 study. J. Hepatol..

[78] Urban S., Bartenschlager R., Kubitz R., Zoulim F. (2014). Strategies to inhibit entry of HBV and HDV into hepatocytes. Gastroenterology.

[79] Petersen J., Dandri M., Mier W., Lutgehetmann M., Volz T., Von Weizsäcker F., Haberkorn U., Fischer L., Pollok J.-M., Erbes B. (2008). Prevention of hepatitis B virus infection in vivo by entry inhibitors derived from the large envelope protein. Nat. Biotechnol..

[80] Lutgehetmann M., Mancke L.V., Volz T., Helbig M., Allweiss L., Bornscheuer T., Pollok J.M., Lohse A.W., Petersen J., Urban S. (2012). Humanized chimeric uPA mouse model for the study of hepatitis B and D virus interactions and preclinical drug evaluation. Hepatology.

[81] Volz T., Allweiss L., Ben Ḿbarek M., Warlich M., Lohse A.W., Pollok J.M., Alexandrov A., Urban S., Petersen J., Lutgehetmann M. (2013). The entry inhibitor Myrcludex-B efficiently blocks intrahepatic virus spreading in humanized mice previously infected with hepatitis B virus. J. Hepatol..

[82] Chen Y., Cheng G., Mahato R.I. (2008). RNAi for treating hepatitis B viral infection. Pharm. Res..

[83] Yuen M.F., Liu K., Chan H.L., Given B.D., Schluep T., Hamilton J., Lai C.L., Locarnini S.A., Lau J.Y., Ferrari C. (2017). Prolonged RNA interference therapy with ARC-520 Injection in treatment naïve, HBeAg positive and negative patients with chronic HBV results in significant reductions of HBs antigen. J. Hepatol..

[84] Arrowhead pharmaceuticals. http://ir.arrowheadpharma.com/static-files/21a89f86-9777-4c2a-8092-af234dd0d4aa.

[85] Janssen H.L.A., Brunetto M.R., Kim Y.J., Ferrari C., Massetto B., Nguyen A.H., Joshi A., Woo J., Lau A.H., Gaggar A. (2018). Safety, efficacy and pharmacodynamics of vesatolimod (GS-9620) in virally suppressed patients with chronic hepatitis B. J. Hepatol..

[86] European Association for the Study of the Liver. https://livertree.easl.eu/easl/2017/international.liver.congress/170380/heiner.wedemeyer.therapeutic.vaccination.of.chronic.hepatitis.b.patients.with.htm.

[87] European Association for the Study of the Liver. https://www.journal-of-hepatology.eu/article/S0168-8278(17)30463-4/pdf.

[88] Rantala M., van de Laar M.J. (2008). Surveillance and epidemiology of hepatitis B and C in Europe—A review. Eurosurveillance.

[89] Duffell E.F., van de Laar M.J. (2015). Survey of surveillance systems and select prevention activities for hepatitis B and C, European Union/European Economic Area, 2009. Eurosurveillance.

[90] WHO Publication (2010). Hepatitis B vaccines: WHO position paper-recommendations. Vaccine.

[91] World Health Organization Global Alert and Response. Hepatitis, B. Prevention and Treatment. http://www.who.int/csr/disease/hepatitis/whocdscsrlyo20022/en/index5.html.

[92] François G., Hallauer J., Van Damme P. (2002). Hepatitis B vaccination: How to reach risk groups. Vaccine.

[93] Rivas I., Martínez E., Sanvisens A., Bolao F., Tor J., Torrens M., Pujol R., Fuster D., Rey-Joly C., Muñoz A. (2010). Hepatitis B virus serum profiles in injection drug users and rates of immunization over time in Barcelona: 1987–2006. Drug Alcohol Depend..

[94] Van Houdt R., Koedijk F., Bruisten S., De Coul E.O., Heijnen M., Waldhober Q., Veldhuijzen I., Richardus J., Schutten M., Van Doornum G. (2009). Hepatitis B vaccination targeted at behavioural risk groups in the Netherlands: Does it work?. Vaccine.

[95] Alter M.J., Hadler S.C., Margolis H.S., Alexander W.J., Hu P.Y., Judson F.N., Mares A., Miller J.K., Moyer L.A. (1990). The changing epidemiology of hepatitis B in the United States. Need for alternative vaccination strategies. JAMA.

[96] Pollard A.J. (2007). Hepatitis B vaccination. BMJ.

[97] World Health Organization. http://www.who.int/immunization/monitoring_surveillance/routine/coverage/en/index4.html.

[98] World Health Organization. http://apps.who.int/iris/bitstream/10665/246177/1/WHO-HIV-2016.06-eng.pdf?ua=1.

[99] Hilleman M.R., Ellis R.W., Marcel D. (1979). Plasma-Derived Hepatitis B Vaccine: A Break-Through in Preventive Medicine.

[100] Sitrin R.D., Wampler D.E., Ellis R.W., Ellis R.W. (1993). Survey of Licensed Hepatitis B Vaccines and Their Production Processes.

[101] Stephenne J. (1998). Development and production aspects of a recombinant yeast-derived hepatitis B vaccine. Vaccine.

[102] Kniskern P.J., Miller W.J. (1992). Hepatitis B vaccines: Blueprints for vaccines of the future. Biotechnology.

[103] Bosnak M., Dikici B., Bosnak V., Haspolat K. (2002). Accelerated hepatitis B vaccination schedule in childhood. Pediatr. Int..

[104] Saltoğlu N., Inal A.S., Tasova Y., Kandemir O. (2003). Comparison of the accelerated and classic vaccination schedules against Hepatitis B: Three-week Hepatitis B vaccination schedule provides immediate and protective immunity. Ann. Clin. Microbiol. Antimicrob..

[105] Jin H., Tan Z., Zhang X., Wang B., Zhao Y., Liu P. (2015). Comparison of Accelerated and Standard Hepatitis B Vaccination Schedules in High-Risk Healthy Adults: A Meta-Analysis of Randomized Controlled Trials. PLoS ONE.

[106] Launay O., van der Vliet D., Rosenberg A.R., Michel M.L., Piroth L., Rey D., Colin de Verdière N., Slama L., Martin K., Lortholary O. (2011). ANRS HB03 VIHVAC-B Trial. Safety and immunogenicity of 4 intramuscular double doses and 4 intradermal low doses vs standard hepatitis B vaccine regimen in adults with HIV-1: A randomized controlled trial. JAMA.

[107] British HIV Association Guidelines on the Use of Vaccines in HIV-Positive Adults. http://www.bhiva.org/documents/Guidelines/Vaccination/2015-Vaccination-Guidelines.pdf.

[108] Lebre F., Borchard G., de Lima M.C., Borges O. (2011). Progress towards a needle-free hepatitis B vaccine. Pharm. Res..

[109] Neutra M.R., Kozlowski P.A. (2006). Mucosal vaccines: The promise and the challenge. Nat. Rev. Immunol..

[110] Holmgren J., Czerkinsky C. (2005). Mucosal immunity and vaccines. Nat. Med..

[111] Vajdy M., O’Hagan D.T. (2001). Microparticles for intranasal immunization. Adv. Drug Deliv. Rev..

[112] Podda A., Del Giudice G. (2003). MF59-adjuvanted vaccines: Increased immunogenicity with an optimal safety profile. Expert Rev. Vaccines.

[113] Bienzle U., Günther M., Neuhaus R., Vandepapeliere P., Vollmar J., Lun A., Neuhaus P. (2003). Immunization with an adjuvant hepatitis B vaccine after liver transplantation for hepatitis B-related disease. Hepatology.

[114] Cooper C.L., Davis H.L., Angel J.B., Morris M.L., Elfer S.M., Seguin I., Krieg A.M., Cameron D.W. (2005). CPG 7909 adjuvant improves hepatitis B virus vaccine seroprotection in antiretroviral-treated HIV-infected adults. AIDS.

[115] Sanyal G., Shi L. (2009). A review of multiple approaches towards an improved hepatitis B vaccine. Expert Opin. Ther. Pat..

[116] Desombere I., Van Der Wielen M., Van Damme P., Stoffel M., De Clercq N., Goilav C., Leroux-Roels G. (2002). Immune response of HLA DQ2 positive subjects, vaccinated with HBsAg/AS04, a hepatitis B vaccine with a novel adjuvant. Vaccine.

[117] Jacques P., Moens G., Desombere I., Dewijngaert J., Leroux-Roels G., Wettendorff M., Thoelen S. (2002). The immunogenicity and reactogenicity profile of a candidate hepatitis B vaccine in an adult vaccine non-responder population. Vaccine.

[118] Milich D., Thornton G., Neurath A., Kent S., Michel M., Tiollais P., Chisari F. (1985). Enhanced immunogenicity of the pre-S region of hepatitis B surface antigen. Science.

[119] CDC (2018). Prevention of hepatitis B virus infection in the United States: Recommendations by the Advisory Committee on Immunization Practices. MMWR Recomm. Rep..

[120] Michel M.L., Pontisso P., Sobczak E., Malpiece Y., Streeck R.E., Tiollais P. (1984). Synthesis in animal cells of hepatitis B surface antigen particles carrying a receptor for polymerized human serum albumin. Proc. Natl. Acad. Sci. USA.

[121] Tron F., Degos F., Bréchot C., Couroucé A.M., Goudeau A., Marie F.N., Adamowicz P., Saliou P., Laplanche A., Benhamou J.P. (1989). Randomized dose range study of a recombinant hepatitis B vaccine produced in mammalian cells and containing the S and PreS2 sequences. J. Infect. Dis..

[122] Raz R., Dagan R., Gallil A., Brill G., Kassis I., Koren R. (1996). Safety and immunogenicity of a novel mammalian cell-derived recombinant hepatitis B vaccine containing Pre-S1 and Pre-S2 antigens in children. Vaccine.

[123] Shapira M.Y., Zeira E., Adler R., Shouval D. (2001). Rapid seroprotection against hepatitis B following the first dose of a Pre-S1/Pre-S2/S vaccine. J. Hepatol..

[124] Gatanaga H., Hayashida T., Tanuma J., Oka S. (2013). Prophylactic Effect of Antiretroviral Therapy on Hepatitis B Virus Infection. Clin. Infect. Dis..

[125] Heuft M.M., Houba S.M., van den Berk G.E.L., Smissaert van de Haere T., van Dam A.P., Dijksman L.M., Regez R.M., Brinkman K. (2014). Protective effect of hepatitis B virus-active antiretroviral therapy against primary hepatitis B virus infection. AIDS.

[126] Falade-Nwulia O., Seaberg E.C., Snider A.E., Rinaldo C.R., Phair J., Witt M.D., Thio C.L. (2015). Incident Hepatitis B Virus Infection in HIV-Infected and HIV-Uninfected Men Who Have Sex with Men from Pre-HAART to HAART Periods A Cohort Study. Ann. Intern. Med..

